# Identification of Genes Associated with Crest Cushion Development in the Chinese Crested Duck

**DOI:** 10.3390/ani12162150

**Published:** 2022-08-22

**Authors:** Qixin Guo, Lan Huang, Yong Jiang, Zhixiu Wang, Guohong Chen, Hao Bai, Guobin Chang

**Affiliations:** 1 College of Animal Science and Technology, Yangzhou University, Yangzhou 225009, China; 2 Joint International Research Laboratory of Agriculture and Agri-Product Safety, The Ministry of Education of China, Yangzhou University, Yangzhou 225009, China

**Keywords:** crested duck, crest cushion, RNA sequence, differentially expressed genes (DEGs), weighted gene co-expression network analysis (WGCNA)

## Abstract

**Simple Summary:**

The crest cushion is a phenotypic appendage observed in most birds, and its appearance differs according to the species. However, the mechanism underlying the formation of the crest cushion and the changes in the expression of the associated genes during development has not been elucidated. We aimed to study the differential expression of selected genes in the Chinese crested duck to assess the genes involved in crest cushion formation and their expression. Our results show that the expression of genes belonging to the homeobox family was significantly altered during the various developmental stages, highlighting their role in the development of the crest cushion. This study elucidated a method to assess the molecular mechanism involved in the formation of the crest cushion and to assess the changes in the gene expression at the genome level.

**Abstract:**

The crest trait is a specific and widely distributed phenotype in birds. However, the shape and function vary in different species of birds. To understand the mechanism of crest formation, the present study used RNA sequencing and weighted gene co-expression network analysis (WGCNA) to identify the crest-cushion-associated genes in the Chinese crested (CC) duck. As a result, 28, 40, 32, 33, and 126 differentially expressed genes (DEGs) were identified between CC and cherry valley (CV) ducks at the embryonic days (E)15, E22, E28, D7 (7 days old), and D42 stages, respectively. In addition, the results of WGCNA show that 3697 (turquoise module), 485 (green-yellow module), 687 (brown module), 205 (red module), and 1070 (yellow module) hub genes were identified in the E15, E22, E28, D7, and D42 stages, respectively. Based on the results of DEGs and WGCNA Venn analysis, three, two, zero, one, and seven genes were found to be associated with crest cushion formation at the E15, E22, E28, D7, and D42 stages, respectively. The expression of all the associated genes and some DEGs was verified by real-time quantitative polymerase chain reaction. In conclusion, this study provided an approach revealing the molecular mechanisms underlying the crested trait development.

## 1. Introduction

Headgear traits, also known as cranial appendages, are common, conspicuous, and varied in animals. Their functions are varied, often stemming from mate selection [1,2], and include feeding, sensing, and even movement assistance [3,4]. In mammals, headgear traits are most commonly found in cloven-hoofed animals, known as “artiodactyles”, such as goats, hippos, cows, and deer [5,6]. The cranial appendages of mammals are generally bony. However, they are widely distributed in birds, such as chickens [7], ducks [8,9], parrots, and pigeons. In birds, cranial appendages are generally derived from feathers.

The Chinese crested (CC) duck is a unique duck breed with spherical feathers and sarcoid protrusions in the skull. According to records, the history of CC ducks can be traced back to the Yuan Dynasty. Furthermore, the crested ducks, Hochbrutflugenten and Landenten, were also discovered by accident in Europe in the 17th and 18th centuries, as recorded by Charles Robert Darwin [10]. The existence of a crest cushion may lead to morphological changes in the skull and brain [11]. In the CC duck, the crest cushion is located posteriorly above the parietal bones and is often underlaid by a mass of fat and connective tissue [9]. Based on histological examination, the crest cushion consists of special high feathers, small holes in the skull, fat tissue, and other small changes that are unknown to date. In addition, some studies on crested chicken and crested ducks have suggested that homeobox c8 (*HOXC8*) plays a critical role in crest cushion formation. In the Beijing-You chicken, a quantitative trait locus (QTL) close to *HOXC8* is significantly associated with the crest phenotype. The homeobox family is a super gene family, and hox genes are a subset of homeobox genes, which are essential metazoan genes that determine the identity of embryonic regions along the anterior–posterior axis [12]. *HoxD3* and *HoxB3* are pro-invasive, angiogenic genes that upregulate b3 and a5 integrins and *Efna1* in endothelial cells (ECs), respectively [13,14]. *HoxA3* induces EC migration by upregulating *MMP14* and *uPAR* [15,16]. Conversely, *HoxD10* and *HoxA5* have the opposite effect of suppressing EC migration and angiogenesis and stabilizing adherens junctions by upregulating *TIMP1*/downregulating *uPAR* and *MMP14* and by upregulating Tsp2/downregulating *VEGFR2*, Efna1, Hif1alpha, and *COX-2*, respectively [17,18]. 

In the present study, we identified genes with differential expression, and then performed weighted gene co-expression network analysis (WGCNA) to screen key candidate genes associated with crest cushion formation to explore gene expression changes in the whole genome during the development of CC ducks and to study the developmental regulation of the crested trait at the genome level. In addition, RT-qPCR was used to verify certain key candidate genes at the mRNA level to provide a molecular basis for elucidating the formation mechanism of the crest trait.

## 2. Materials and Methods

### 2.1. Ethical Approval

All experiments on chickens and eggs were performed in accordance with the Regulations on the Administration of Experimental Animals issued by the Ministry of Science and Technology (Beijing, China) in 1988 (last modified in 2001). The experimental protocols were approved by the Animal Care and Use Committee of the Yangzhou University (YZUDWSY2017-11-07). All efforts were made to minimize animal suffering.

### 2.2. Experimental Animals and Sample Collection

Samples of the crest tissue were collected from 30 CC ducks from five developmental stages (embryonic day (E) 15, E22, E28, D7 (7 days old), and D42 (42 days old)). Samples of scalp tissue were also collected from 30 CV ducks at the same five developmental stages as the CC ducks (Figure S1). All eggs and ducks in the experiment were incubated in the same incubation conditions and in the same rearing environment in Zhenjiang Tiancheng Agricultural Technology Co., Ltd. Six animals (for both CC and CV ducks) were sampled for each developmental stage. Three of the six samples were randomly selected for RNA seq. The samples were immediately frozen in liquid nitrogen and stored at −80 °C prior to RNA isolation. Total RNA was extracted from skin samples using RNA Simple Total RNA Kit (Cat. no. DP419, Beijing, China) according to the manufacturer’s instructions. RNA degradation and contamination were monitored using 1% agarose gels, and RNA purity was evaluated using a NanoPhotometer spectrophotometer (IMPLEN, Westlake Village, CA, USA). RNA concentration was measured using a Qubit^®^ RNA Assay Kit with a Qubit^®^ 2.0 Fluorometer (Life Technologies, Carlsbad, CA, USA). RNA integrity was assessed using an RNA Nano 6000 Assay Kit and an Agilent 2100 Bioanalyzer system (Agilent Technologies, Santa Clara, CA, USA).

### 2.3. Library Construction and Sequencing

A total of 3 μg of RNA per sample was used for transcriptome sequencing. The PCR products were purified using the AMPure XP system, and library quality was assessed using the Agilent 2100 Bioanalyzer system. After cluster generation, the library was sequenced using an Illumina NovaSeq 6000 platform by Novogene Biotechnology Co., Ltd. (Beijing, China), and 150 bp paired-end reads were generated. The quality of the RNA sequences was checked using FastQC, and sequence adapters and low-quality reads (read quality < 20) were removed using N 0.001 L 20 p 0.5. Sequenced reads were mapped to the duck genome (unpublished data) assembled in our laboratory using HISAT v2.1.0. To quantify the expression of each transcript, alignment results were analyzed using the FeatureCounts (1.5.0-p3) software (-Q 10 -B -C).

### 2.4. Analysis of Differentially Expressed Genes (DEGs) 

The software FeatureCounts was used to analyze gene abundance, and the expression level was normalized to the number of fragments per kilobase of transcript read per million mapped reads. The cpm function converts raw counts into CPM and log-CPM values. The trimmed mean of M values (TMM) in the edgeR package was used to normalize the read count. The R/edgeR package was used to identify DEGs. The primary parameters, false discovery rate (FDR), log fold change (log-FC), log counts per million (log-CPM), and *p*-values, were used to determine the DEGs. Volcano plots and heatmaps were used for visualization.

### 2.5. WGCNA

The R/WGCNA package was used for coexpression analysis. A total of 17,425 genes and 30 samples with values exceeding the fragments per kilobase of transcript per million mapped reads (FPKM) value were used to construct the coexpression network. Two samples were removed based on hclust, which was used to construct a sample tree to predict outlier samples. Additionally, 13,068 genes passed the missed-value detection. A soft thresholding power of β = 5 (R^2^ > 0.9) was used in the present study. The settings corType = ‘pearson’, mergeCutHeight = 0.25, and minModuleSize = 30 were used to automatically construct the network and define gene modules by blockwise modules. Module membership (MM) was defined as the correlation between eigengenes in a given module and the gene expression profile. Modules satisfying the conditions of |GS versus MM| > median and *p*-value < 0.01 are known to be highly related to stage and trait. Intramodular hub genes in interesting modules were detected as genes highly connected to high module membership and were highly correlated with the crested cushion developmental stage. 

### 2.6. Real-Time Quantitative Polymerase Chain (RT-qPCR)

To validate the accuracy of the RNA sequencing data, RT-qPCR was performed to visualize the DEGs and hub genes. Total RNA was extracted using the RNA Simple Total RNA Kit (Cat. no. DP419, Beijing, China), according to the manufacturer’s protocol. One microgram of total RNA was reverse-transcribed to cDNA using a Fast Quant RT Kit (with gDNase) (Tiangen, Beijing, China). The QuantStudio™ 5 Real-Time PCR System was used to detect gene expression, and gene-specific primer cDNAs are shown in Table S1. The cycling conditions were as follows: 95 °C for 5 min, followed by 40 cycles of 95 °C for 10 s, and 60 °C for 30 s. The Ct values were obtained using default settings, and the relative mRNA expression of the target genes was calculated using the 2^−ΔΔ^Ct method after normalization to the levels of *GAPDH* as the internal control. All samples were analyzed in triplicate. 

### 2.7. Statistical Analysis

All results displayed in the bar graphs are expressed as the mean ± standard error of the mean (SEM) of three independent experiments. Statistical significance was determined using Student’s *t*-test in the R/stats package. Statistical significance was set at *p* < 0.05.

## 3. Results

### 3.1. Overview of RNA Sequencing Data

**Figure 1:**
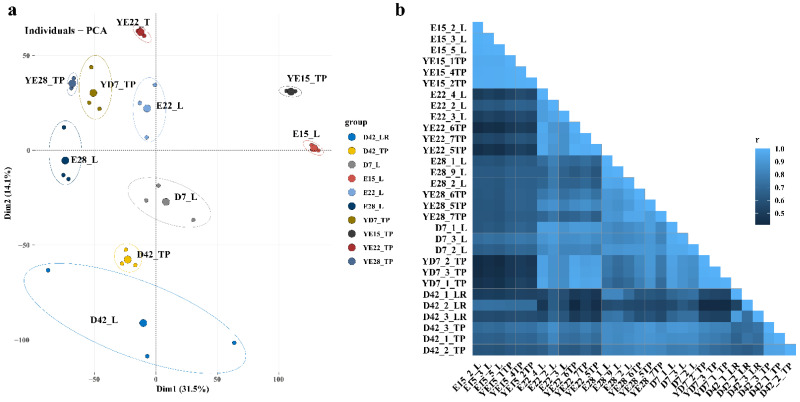
Sample correlation analysis. (**a**) PCA analysis of all RNA sequencing samples; (**b**) heat map of the correlation of all RNA sequencing samples. The shades of colors represent different correlation coefficients (r).

To identify the DEGs in the five different developmental stages, RNAs isolated from two different duck breeds (three replicates for each developmental stage for each breed) at the E15, E22, E28, D7, and D42 stages were selected to construct 30 cDNA libraries denoted as follows: E15_L for crest cushion from CC duck at the E15 stage; E22_L for crest cushion from CC duck at the E22 stage; E28_L for crest cushion from CC duck at the E28 stage; D7_L for crest cushion from CC duck at the D7 stage; D42_L for crest cushion from CC duck at the D42 stage; YE15_TP for scalp tissue from CV duck at the E15 stage; YE22_TP for scalp tissue from CV duck at the E22 stage; YE28_TP for scalp tissue from CV duck at the E28 stage; YD7_TP for scalp tissue from CV duck at the D7 stage; and D42_TP for scalp tissue from CV duck at the D42 stage. Among the high-quality clean reads generated from the 30 samples, the percentage of total mapped reads was 83.37–88.01% (Table S1). To determine the relationship among the three biological replicates, we clustered the samples using principal component analysis (PCA) and correlation analysis. The PCA and correlation heatmap results show excellent reproducibility among the biological replicates (Figure 1a,b). The Pearson correlation coefficient for the three biological replicates of each group also indicated a very high reproducibility (R^2^ ≥ 0.91 for all).

### 3.2. Differential Gene Expression Analysis in Each Stage

**Figure 2:**
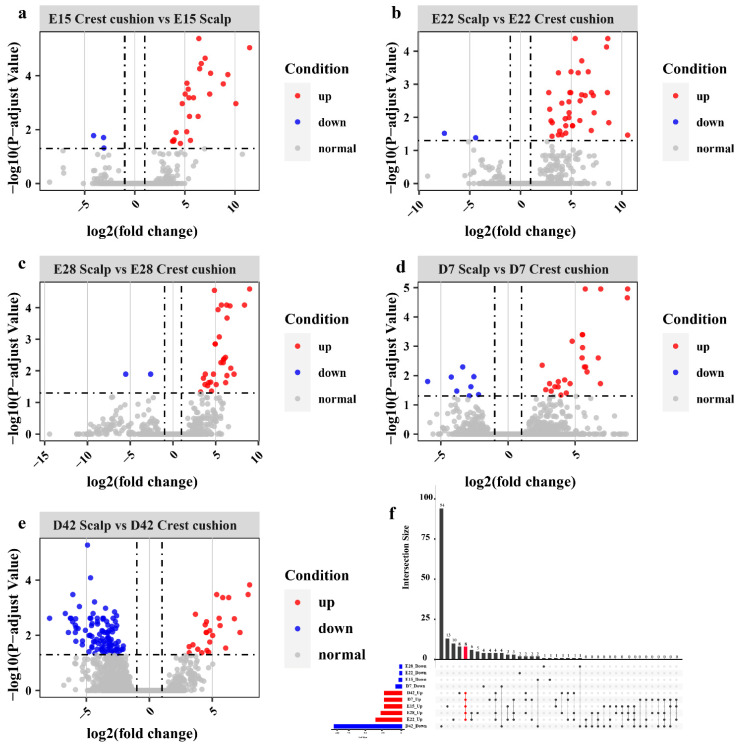
Volcano plots of the distribution trends of differentially expressed genes in crest cushion and scalp tissue at different developmental stages. (**a**) E15 stage; (**b**) E22 stage; (**c**) E28 stage; (**d**) D7 stage; (**e**) D42 stage; (**f**) Upset plot of all comparison groups.

To identify the genes with differential expression between the crest cushion and scalp tissue, the R/edgeR package was used to determine the DEGs using the following criteria: FDR ≤ 0.05 and logFC > 1 (Table S2). Among the E15_L and YE15_TP comparison groups, 28 DEGs (3 downregulated and 25 upregulated) were identified (Figure 2a). A total of 40 DEGs (2 downregulated and 38 upregulated) were identified among the E22_L and YE22_TP comparison groups (Figure 2b), and 32 DEGs (2 downregulated and 30 upregulated) were identified among the E28_L and YE28_TP comparison groups (Figure 2c). Among the D7_L and YD7_TP comparison groups, 33 DEGs (8 downregulated and 25 upregulated) were identified (Figure 2d). A total of 126 DEGs (101 downregulated and 25 upregulated) were identified between the D42_L and YD42_TP comparison groups (Figure 2e). Among all the comparison groups, a total of eight genes (*HOXC10*, *HOXA6*, *HOXC9*, *HOXC6*, *HOXA10*, *HOXC10*, *HOXA3* and *HOXB5*), all belonging to the homeobox gene family, were commonly upregulated in the crest cushion in each developmental stage (Figure 2f).

### 3.3. Hub Genes Identified in the Crest Cushion by Weighted Coexpression Network Analysis

**Figure 3:**
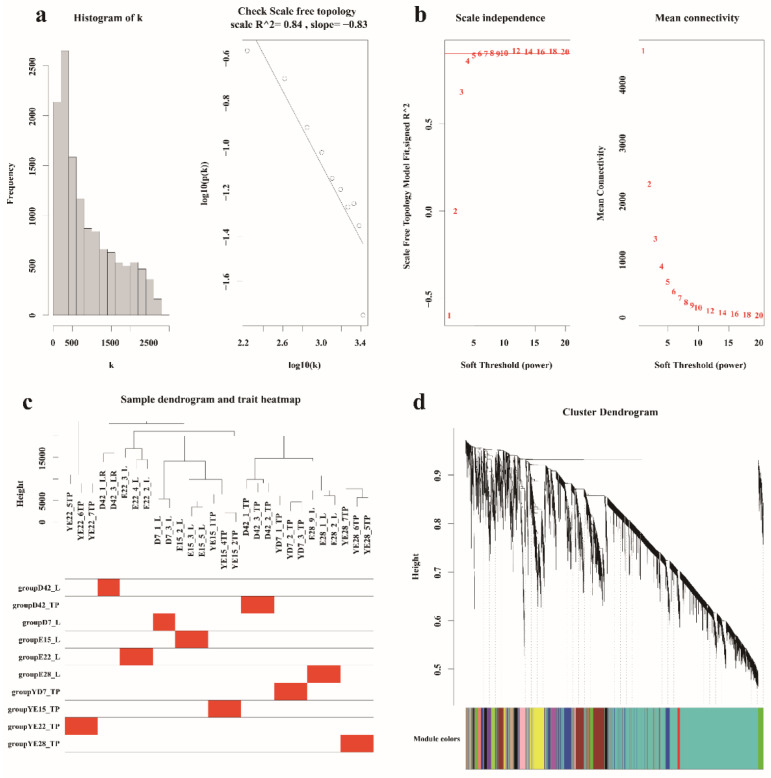
Weighted gene coexpression network analysis of all samples. (**a**) Histogram showing k and the correlation coefficient between k and *p* (k) for all samples. (**b**) The scale independence and the mean connectivity of the WGCNA analysis of all samples. (**c**) The clustering dendrogram of samples to detect outliers. (**d**) Clustering dendrograms of all samples.

**Figure 4:**
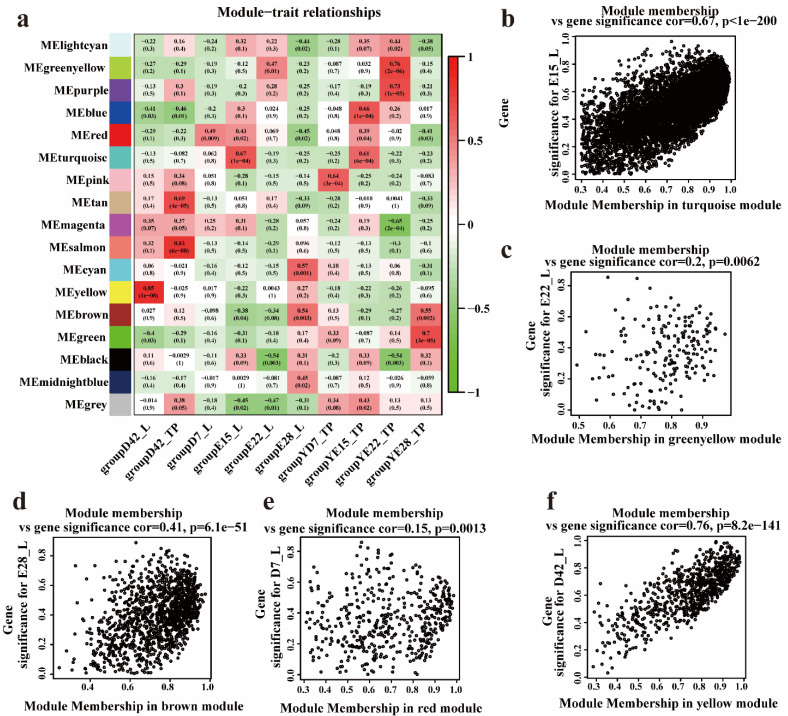
Hub genes in different developmental stages of crest cushion: (**a**) the module–trait relationship; (**b**) module membership and gene significance correlation in turquoise module; (**c**) module membership and gene significance correlation in greenyellow module; (**d**) module membership and gene significance correlation in brown module; (**e**) module membership and gene significance correlation in red module; (**f**) module membership and gene significance correlation in yellow module.

To identify key genes related to each trait at the different developmental stages, WGCNA was performed to correlate each module with the appropriate traits. After preprocessing the data, data quality was evaluated by sample clustering based on the distance between different samples observed in Pearson’s correlation matrices. To eliminate outliers, we removed a sample (D42_2_L, D7_2_L) based on a hierarchical clustering tree (dendrogram). Next, we chose the power of β = 5 (scale-free R^2^ = 0.84, slope = −0.83) as the soft-thresholding parameter to construct a scale-free network (Figure 3a–c). As a result, 13,068 genes were grouped into a total of 15 modules based on the dynamic tree-cutting algorithm (Figure 3d), namely turquoise [6565], black [409], blue [1379], brown [1227], cyan [91], green [597], green-yellow [186], grey [308], light-cyan [59], magenta [224], midnight-blue [83], pink [251], purple [187], red [454], salmon [126], tan [179], and yellow modules [743] (Figure 4a). The module eigengenes (MEs) in the yellow module (R^2^ = 0.85, *p* = 1 × 10^−8^) showed a higher correlation with D42_L; those in the red (R^2^ = 0.49, *p* = 0.009) module showed a higher correlation with D7_L; and those in the cyan module (R^2^ = 0.57, *p* = 0.001) showed a higher correlation with E28_L, whereas those in the green-yellow module (R^2^ = 0.47, *p* = 0.01) showed a higher correlation with E22_L, and those in the turquoise module (R^2^ = 0.67, *p* = 1 × 10^−4^) showed a higher correlation with the E15 stage (Figure 4a). These modules were used for further research.

### 3.4. Identifying Hub Genes Associated with the Crest Cushion in the Different Developmental Stages

To detect significant relationships between the genes and phenotypes, trait–module relationships were evaluated by correlating the module–sample eigengenes with the crest cushion. To distinguish between the modules, the colors of each module were randomly selected. Consequently, the turquoise (cor = 0.67, *p* < 1 × 10^−200^) module exhibited a positive correlation with the crest cushion at the E15 stage (Figure 4b). The green-yellow module was correlated with the crest cushion at the E22 stage (cor = 0.2, *p* < 0.0062) (Figure 4c). The brown module was correlated with the crest cushion at the E28 stage (cor = 0.41, *p* < 6.1 × 10^−51^) (Figure 4d). The red module was significantly correlated with the crest cushion at the D7 stage (cor = 0.15, *p* < 0.0013) (Figure 4e). The yellow module was significantly correlated with the crest cushion at the D42 stage (cor = 0.76, *p* < 8.2 × 10^−41^) (Figure 4f). Furthermore, under the condition of higher-than-median MM and gene significance (GS), 3697 genes in the turquoise module (median GS ≥ 0.36, range = 1.41 × 10^−5^–0.964, median MM ≥ 0.554, range = 8.191 × 10^−5^–0.9916873, and GS *p*-value ≤ 0.01) were retained for the identified crest cushion trait at the E15 stage. In addition, 485 genes in the green-yellow module (median GS ≥ 0.176, range = 2.079 × 10^−5^–0.936, median MM ≥ 0.239, range = 4.251 × 10^−5^–0.973, and GS *p*-value ≤ 0.01) were identified as hub genes of the crest cushion at the E22 stage based on the MM and GS. A total of 687 genes in the brown module were identified as hub genes of the crest cushion at the E28 stage (with median GS ≥ 0.222, range = 5.618 × 10^−5^–0.889, median MM ≥ 0.422, range = 1.692 × 10^−5^–0.976, and GS *p*-value ≤ 0.01). Meanwhile, 205 genes in the red module were identified as hub genes of the crest cushion at the D7 stage (with median GS ≥ 0.134, range = 1.478 × 10^−5^–0.857, median MM ≥ 0.454, range = 6.272 × 10^−5^–0.985, and GS *p*-value ≤ 0.01). A total of 1070 genes in the yellow module were identified as hub genes of the crest cushion at the D42 stage (with median GS ≥ 0.19, range = 4.368 × 10^−5^–0.992, median MM ≥ 0.291, range = 2.138 × 10^−5^–0.980, and GS *p*-value ≤ 0.01).

### 3.5. Validation of Genes Associated with the Crest Cushion 

**Figure 5:**
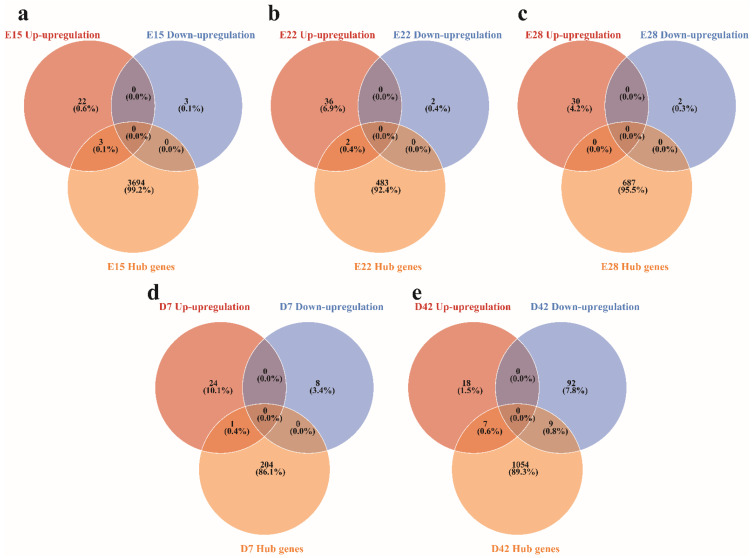
Genes associated with crest cushion in the different developmental stages. Venn analysis of DEGs and hub genes in the E15 (**a**), E22 (**b**), E28 (**c**), D7 (**d**), and D42 (**e**) stages.

**Figure 6:**
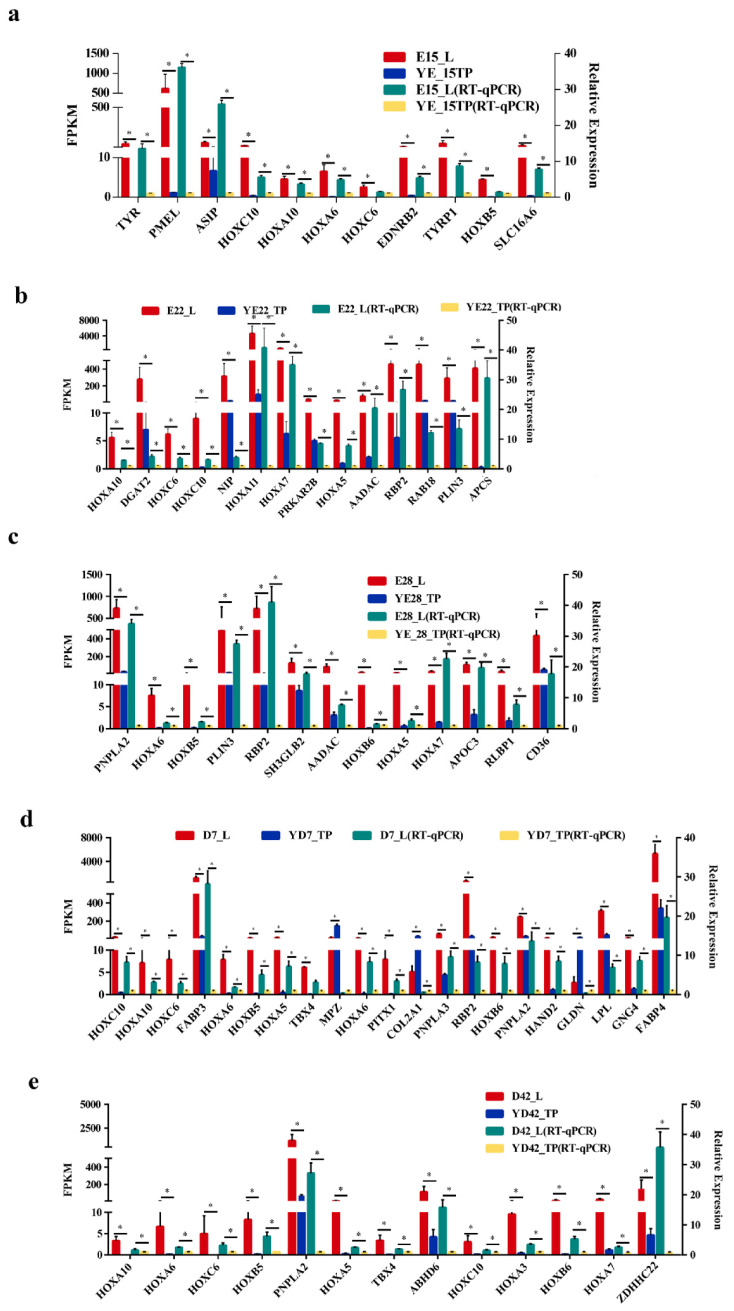
Validation of genes associated with crest cushion. Expression level of genes associated with crest cushion at the E15 (**a**), E22 (**b**), E28 (**c**), D7 (**d**), and D42 (**e**) stages. * Represents significant difference (*p*-value < 0.05).

To determine genes associated with the crest cushion, we used Venn diagrams to analyze the DEGs and hub genes at each crest cushion developmental stage. The results show that three genes were highly associated with crest cushion at the E15 stage (Figure 5a), two genes were associated with crest cushion at the E22 stage (Figure 5b), zero gene which overlapped with DEGs and hub genes at the E28 stage (Figure 5c), one gene was associated with crest cushion at the D7 stage (Figure 5d), and seven genes were associated with crest cushion at the D42 stage (Figure 5e). We also validated the expression of multiple genes at each stage. The results show that almost all genes belonging to the homeobox gene family were differentially expressed between the crest cushion and scalp tissues at different embryonic developmental stages (Figure 6).

## 4. Discussion

During species evolution, directional artificial selection and non-directional natural selection can cause genetic diversity in animals. The crest cushion trait is the result of a long period natural selection. The crested head, a phenotype unique to the CC duck, is similar to the crown of a chicken, the top hair of a peacock, the crest of a crested zoanthid, etc. In contrast to these phenotypes, however, the crested duck’s crested head has some special features, such as cranial skin protrusions, incomplete cranial closure, intracranial fatty tissue, and rounded feathers. The crest cushion is specific to CC ducks among all the duck breeds in China. Previous studies by our group have shown that a distinct protrusion forms at the top of the skull in E4 crested duck embryos. At E15, however, the cranial cartilage was found to be incompletely closed, and the cranial protrusion was significantly larger at this time. Before emergence, we found that the cranial bones were still incompletely closed and that the cranial prominence had a flesh-like appearance with soft tissue growth. Coincidentally, the preadipocytes begin to differentiate into adipocytes. To compensate for the decrease in cerebral pressure caused by the perforation, different volumes of fat are deposited between the brain and the cerebellum. However, spherical feathers are only used to prevent the fragile epidermis from proliferating. Thus, the formation of the crestal pad is the result of several successive coincidences during the development of the skull, scalp and feathers. Protrusions are probably the most fundamental cause of crown formation [9]. To explore the mechanism of crest cushion formation, DEGs and WGCNA were used to identify genes associated with crest cushion formation in the present study. Among the identified DEGs, 28 (3 downregulated and 25 upregulated), 40 (2 downregulated and 38 upregulated), 32 (2 downregulated and 30 upregulated), 33 (8 downregulated and 25 upregulated), and 126 (101 downregulated and 25 upregulated) were screened between CC and CV ducks at the E15, E22, E28, D7, and D42 stages, respectively. In addition, 3697 hub genes (turquoise module), 485 hub genes (green-yellow module), 687 hub genes (brown module), 205 hub genes (red module), and 1070 hub genes (yellow module) were identified at the E15, E22, E28, D7, and D42 stages, respectively, by WGCNA. The RT-qPCR verification results for upregulated genes in each stage were consistent with those of RNA sequencing. The results show that genes belonging to the homeobox gene family exhibited differential expression between the crest cushion and scalp tissue. Previous studies on crested chicken and ducks suggested that *Hoxc8* is a candidate gene for the regulation of crest cushion formation [19,20]. However, Hoxc8 was not differentially expressed in the present study, but other homeobox genes, such as *HOXA6*, *HOXB5*, *HOXC6*, *HOXA5*, *HOXA7*, and *HOXC10*, showed differential expression. *Hoxb5* binds to the cis-acting element in the first intron of vascular endothelial growth factor receptor-2, which is the earliest marker of endothelial precursor cells. Consistently, *Hoxb5* overexpression in embryonic stem cells results in increased numbers of endothelial precursor cells [21,22]. *Hoxa10* is a transcription factor that regulates gene expression, morphogenesis, and differentiation [23]. Homeobox genes play a major role in specifying segmental differences within the body of vertebrates, which affect morphogenesis and development. Thus, by combining the results of the DEG analysis, it can be inferred that the homeobox genes may play a crucial role in crest cushion formation and development. Further, result of present study suggest that the homeobox gene family may significantly regulate the crest cushion formation. However, the specific molecular mechanism involved in the formation of the crested tissue needs further analysis. 

## 5. Conclusions

In summary, we identified 28, 40, 32, 33, and 126 DEGs between CC and CV ducks at the E15, E22, E28, D7, and D42 stages, respectively, by RNA sequence analysis. In addition, 3697 hub genes (turquoise module), 485 hub genes (green-yellow module), 687 hub genes (brown module), 205 hub genes (red module), and 1070 hub genes (yellow module) were identified in E15, E22, E28, D7, and D42 stages, respectively, by WGCNA. Venn analysis of the results of DEGs and WGCNA showed that 3, 2, 0, 1, and 7 genes were associated with crest cushion at the E15, E22, E28, D7, and D42 stages, respectively. According to the results of the present study, the homeobox gene family may regulate the crest cushion of CC duck formation. The present study has revealed to some extent the changes in gene expression during crest formation, but the regulatory relationship of these genes in the mechanism of crest pad formation is still unclear, With the progress of cell biology, this issue will be gradually resolved in the future.

## Data Availability

The data presented in this study are available in Table S2.

## Supplementary Materials

The following are available online at https://www.mdpi.com/article/10.3390/ani12162150/s1. Figure S1. The pattern diagram of the sample; Table S1: Information regarding the specific primers used for RT-qPCR; Table S2. The FPKM of all genes in each sample.
