# Supplementary material for: Identification of Genes Associated with Crest Cushion Development in the Chinese Crested Duck
Source: Animals (Basel). 2022 Aug 22;12(16):2150. doi: 10.3390/ani12162150 (PMC9404885; doi:10.3390/ani12162150)
Supplement: Supplementary file 1 [file animals-12-02150-s001.zip › Figure S1.pdf]

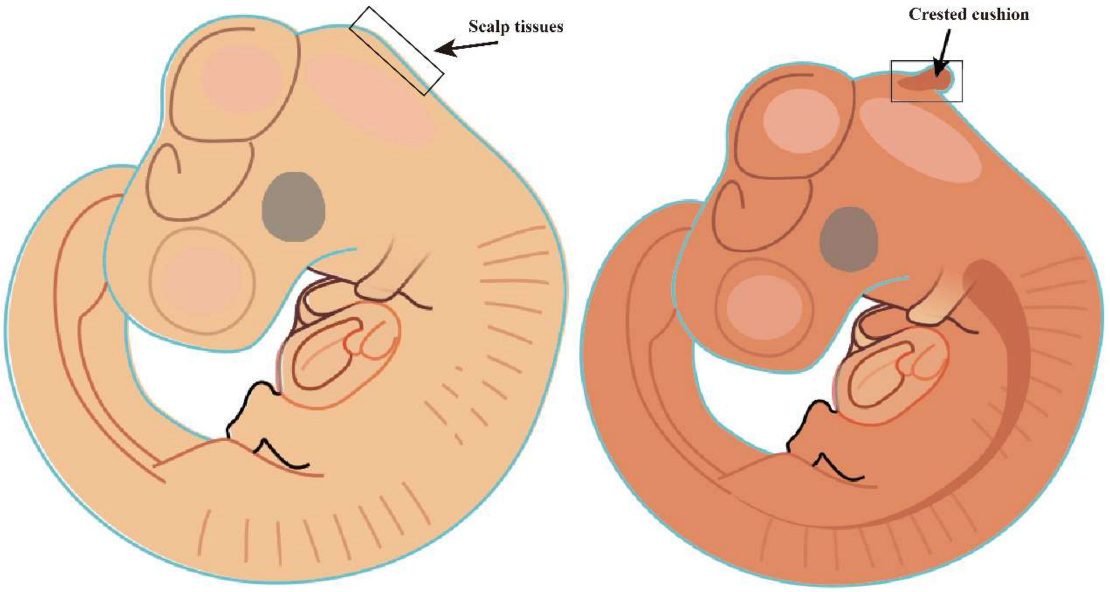


**Figure S1.** The Pattern diagram of the sample. The right pattern diagram represents a normal duck embryo with the area of scalp tissue in the black box. The pattern diagram on the left represents a Chinese crested duck embryo with the area of crest tissue in the black box.
